# Agroinfiltration as an Effective and Scalable Strategy of Gene Delivery for Production of Pharmaceutical Proteins

**DOI:** 10.4172/atbm.1000103

**Published:** 2013-06

**Authors:** Qiang Chen, Huafang Lai, Jonathan Hurtado, Jake Stahnke, Kahlin Leuzinger, Matthew Dent

**Affiliations:** 1 The Biodesign Institute, Center for Infectious Disease and Vaccinology, Arizona State University, USA; 2 College of Technology and Innovation, Arizona State University, USA

**Keywords:** Gene delivery, Agroinfiltration, Plant infiltration, Plant-made pharmaceuticals, Syringe agroinfiltration, Vacuum agroinfiltration, Vaccines, Monoclonal antibody, *Agrobacterium tumefaciens*, *Nicotiana benthamiana*, Geminiviral vectors

## Abstract

Current human biologics are most commonly produced by mammalian cell culture-based fermentation technologies. However, its limited scalability and high cost prevent this platform from meeting the ever increasing global demand. Plants offer a novel alternative system for the production of pharmaceutical proteins that is more scalable, cost-effective, and safer than current expression paradigms. The recent development of deconstructed virus-based vectors has allowed rapid and high-level transient expression of recombinant proteins, and in turn, provided a preferred plant based production platform. One of the remaining challenges for the commercial application of this platform was the lack of a scalable technology to deliver the transgene into plant cells. Therefore, this review focuses on the development of an effective and scalable technology for gene delivery in plants. Direct and indirect gene delivery strategies for plant cells are first presented, and the two major gene delivery technologies based on agroinfiltration are subsequently discussed. Furthermore, the advantages of syringe and vacuum infiltration as gene delivery methodologies are extensively discussed, in context of their applications and scalability for commercial production of human pharmaceutical proteins in plants. The important steps and critical parameters for the successful implementation of these strategies are also detailed in the review. Overall, agroinfiltration based on syringe and vacuum infiltration provides an efficient, robust and scalable gene-delivery technology for the transient expression of recombinant proteins in plants. The development of this technology will greatly facilitate the realization of plant transient expression systems as a premier platform for commercial production of pharmaceutical proteins.

## Introduction

Plants have been explored as alternatives to mammalian, insect, and bacterial cell cultures for the expression and production of recombinant pharmaceutical proteins [1,2]. The recent development of “deconstructed” viral vectors and novel downstream processing regimes has created the potential for plant-based systems to alter the current paradigm of protein production [3]. The promise of this platform has been demonstrated by the recent approval of the first plant-derived therapeutic enzyme for Gaucher’s Disease and the success of several human clinical trials with plant-made pharmaceuticals [4]. Plants offer several advantages over current bioreactor-based platforms for protein production. For example, plant-made biologics cost significantly less than current cell culture-based systems because mammalian cell cultures require considerable startup investment and expensive growth media [5]. Plants also outpace the scalability of other expression systems, as recombinant protein-expressing biomass can be produced on an agricultural scale without the need to build duplicated bioreactors and associated facilities [2]. In contrast to bacterial cells, plants can produce large functional pharmaceutical proteins that require the proper post-translational modification of proteins, including glycosylation and the assembly of multiple heterosubunits [6]. This is possible because plant cells utilize a eukaryotic endomembrane system that is similar to mammalian cells [7]. Furthermore, plant-based expression systems also offer superior benefits to public safety as the risk of transmitting human or animal pathogens from the protein-producing host to humans is greatly reduced [8]. In addition to these traditional advantages, recent progress in plant glycoengineering and expression vector discovery has also allowed the production of human biologics with specific glycoforms for better efficacy and at unprecedented speed to control potential pandemics [4,8,9]. Recombinant proteins can be expressed in plants through two general approaches: by creating stable transgenic plants or by performing transient expression. In the first strategy, the transgene is cloned into an expression vector and delivered to plant genomes. Transgenic plants with the transgene stably integrated into their nuclear or chloroplast genome are generated and selected. As a result, the transgene becomes inheritable through succeeding generations, and transgenic seeds can be stocked for large scale manufacturing of the target protein [1,10]. In transient expression, instead of integrating into the plant genome, the delivered transgene quickly directs the transient production of the desired protein. This strategy offers the advantage of a much improved protein accumulation level, as well as faster protein production timelines [11]. Typically, high levels of recombinant proteins can be recovered from plant tissue approximately 1–2 weeks after gene delivery [12]. Thus, transient expression significantly shortens the timeline of protein production as the processes of generating and selecting stable transgenic plants can take up to a year. However, a scale-up operation of the transient expression system may not appear as straightforward as systems based on transgenic plants, as it will not generate a genetically stable seed bank that can be used to plant on a larger land area when scale-up is needed. Therefore, one critical issue is the need to develop an efficient transgene delivery method that can be used in large-scale transient expression operations.

## Delivery of Transgene into Plant Cells

Regardless of the method of choice for pharmaceutical protein production, the target transgene must be efficiently delivered into host cells. The most common methods of gene introduction into plant cells are direct delivery by biolistics or indirect delivery by using *Agrobacterium tumefaciens* [13]. Biolistics, also known as microprojectile bombardment, involves the coating of gold or tungsten particles (~ 2 microns in diameter) with the transgene and firing them ballistically into plant cells [14]. This method can be used to introduce exogenous DNA to both nuclear and chloroplast genomes and, at least in theory, can be used to transform any plant species and their subcellular organelles [13]. In addition, biolistics do not require specific vectors, thus greatly simplifying the cloning process. Indirect gene delivery methods rely on *Agrobacteria* species that naturally transfer DNA into plant cells. For example, *A. tumefaciens* is a plant pathogen that transfers part of its tumor inducing (Ti) plasmid, called transfer DNA (T-DNA), into plant hosts. As the tumor inducing genes in the T-DNA can be replaced by the gene of interest, the modified Ti plasmids have become the most useful vectors for gene delivery through the natural interaction between *A. tumefaciens* and its plant hosts [13]. Compared with biolistics methods, gene delivery by *A. tumefaciens* is feasible for most dicotyledonous and a limited number of monocotyledonous plant species [15]. Long-term studies have demonstrated that gene delivery methods based on *Agrobacterium* perform significantly better than biolistics in terms of transformation efficiency, transgene expression, and inheritance [13]. These differences may be due to the high transgene copy numbers and the total randomness of gene integration into plant genome as a result of biolistics. In contrast, the coevolution of *Agrobacterium* and its plant hosts may allow for a more precise and selective transgene insertion into the plant genome, which leads to its stable integration and inheritance, and in turn, its consistent expression over generations [15]. Both biolistics and *Agrobacterium-*based methods have been successfully used for generating stable transgenic lines and transient gene expression of many plant species [13]. However, gene delivery by *Agrobacterium* is more desirable than biolistics for the application of pharmaceutical protein production by transient systems, as the latter method routinely results in severe tissue damage and effectively reduces the amount of biomass available for protein production. In the last 20 years, a variety of methods have been developed for *Agrobacterium*-based gene delivery into plant cells [15]. Recently, one method called agroinfiltration has gained momentum as the most promising technology for such applications [16].

## Agroinfiltration Methodologies and Applications

**Figure 1:**
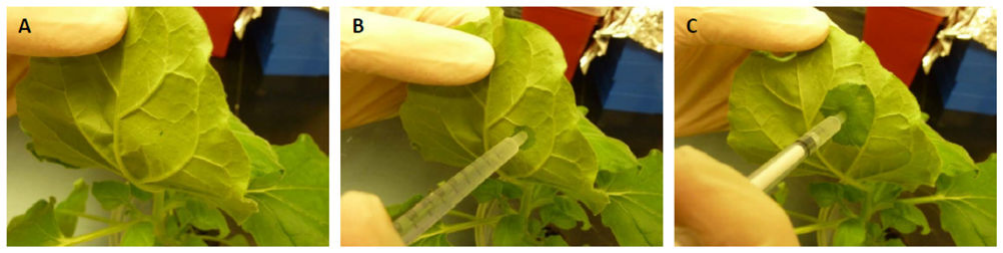
Syringe agroinfiltration of *N. benthamiana* leaves with *Agrobacterium tumefaciens. A. tumefaciens* harboring the gene of interest was resuspended in infiltration buffer and loaded into a syringe without a needle. A nick was created with a needle on the backside of a 6-week old plant leaf (A). *Agrobacteria* were injected into the interstitial space of the leaf via the nick (B and C) [39].

Agroinfiltration was initially developed as a tool to investigate plant-virus interactions. In earlier experiments, whole or partial genomes of plant viruses were cloned into binary vectors and transformed into an *Agrobacterium* host. These exogenous DNA-carrying *Agrobacteria* were then infiltrated into the intercellular space of the plant tissue, allowing the delivery of viral genes into plant genomes [17]. Since then, DNA of interest from different organisms has been delivered into plant cells through agroinfiltration for a broad range of applications far beyond studying plant-virus interactions [16]. The most popular method of agroinfiltration is “syringe agroinfiltration”, involving the use of a needleless syringe to introduce *Agrobacterium* into plant leaves [18]. First, a small nick is created with a needle in the epidermis on the back side of the leaf by gently scratching without piercing through both sides (Figure 1A). *Agrobacterium* in the infiltration medium is then injected into the leaf through the nick with a needleless syringe (Figure 1B). As the *Agrobacterium* mixture enters the intercellular space of the leaf, the light green colour begins to darken, indicating a successful infiltration (Figure 1C). Syringe infiltration has been optimized for several plant species [19] and has demonstrated several critical advantages. It is a simple procedure without the need for any specialized equipment. Moreover, it has the flexibility of either infiltrating the whole leaf with one target DNA construct or introducing multiple constructs into different areas of one leaf, permitting multiple assays to be performed on a single leaf [16]. These advantages have allowed syringe infiltration to become the method of choice for transient gene expression in a broad range of applications including studies of plant pathogen interactions, abiotic stresses, plant gene functional analysis with transient silencing assay, protein localization and function, and protein-protein interactions [16]. Entire leaf syringe agroinfiltration can be applied to obtain laboratory scale of recombinant proteins for their biochemical characterization, purification and preclinical functional studies [1,20,21]. The simplicity of syringe infiltration may also allow it to become a favorable tool in high school and college education for the introduction of genetic engineering. An alternative agroinfiltration method based on the use of a vacuum chamber was first developed for plant species that cannot be efficiently agroinfiltrated by syringe infiltration [13]. In vacuum infiltration, plant leaves are first submerged into an infiltration media which contains the *Agrobacterium* strain harboring the target recombinant DNA. The submerged plants are then subject to a negative atmospheric pressure in a vacuum chamber. The vacuum draws the air out of the interstitial spaces of the submerged plant leaf. Agroinfiltration is then achieved as the space once occupied by air is filled by *Agrobacterium*-containing media when the vacuum is released. Compared to syringe infiltration, vacuum infiltration is more complicated and requires the investment of vacuum pumps, vacuum chambers, and larger volumes of *Agrobacterium* cultures. Furthermore, it loses the flexibility of conducting multiple assays on a single leaf. However, the significance of vacuum infiltration lies far beyond its ability to extend gene and protein functional analyses to plant species that are not amenable to syringe infiltration. Indeed, its value lies in its enormous scalability potential that cannot be matched by syringe infiltration. As discussed later in this review, vacuum infiltration is more robust and can infiltrate a large number of plants in a short period of time. This advantage has facilitated the development of a highly scalable protein production platform for the fast, economical and safe manufacturing of human pharmaceuticals in plants.

## Agroinfiltration for Expression of Pharmaceutical Proteins

One of the early observations of transient assays with agroinfiltration was the high levels of the transgene expression in comparison to that of stable transgenic plants [17]. This is mostly due to the elimination of the “position effect” in the transient expression systems, as the transgene is not randomly inserted into areas of the plant nuclear genome with uncertain transcriptional activities [1,2]. As discussed earlier, the short timeline and higher levels of recombinant protein accumulation make transient expression an attractive platform for pharmaceutical production in plants. However, the scalability of transient expression systems especially that associated with agroinfiltration remained a challenge. Since the production scale could potentially be limited, further optimization of expression vectors was also desirable to achieve even higher levels of protein production. As a result, substantial efforts have been focused on the development of efficient transgene delivery methods that are scalable for large-scale transient expression operations, as well as on the search for new expression vectors to further enhance the accumulation of recombinant protein. Expression vector construction, plant growth, *Agrobacterium* culture preparation, and the actual infiltration are the four major steps of agroinfiltration for the production of pharmaceutical proteins.

## Vector Construction

**Figure 2:**
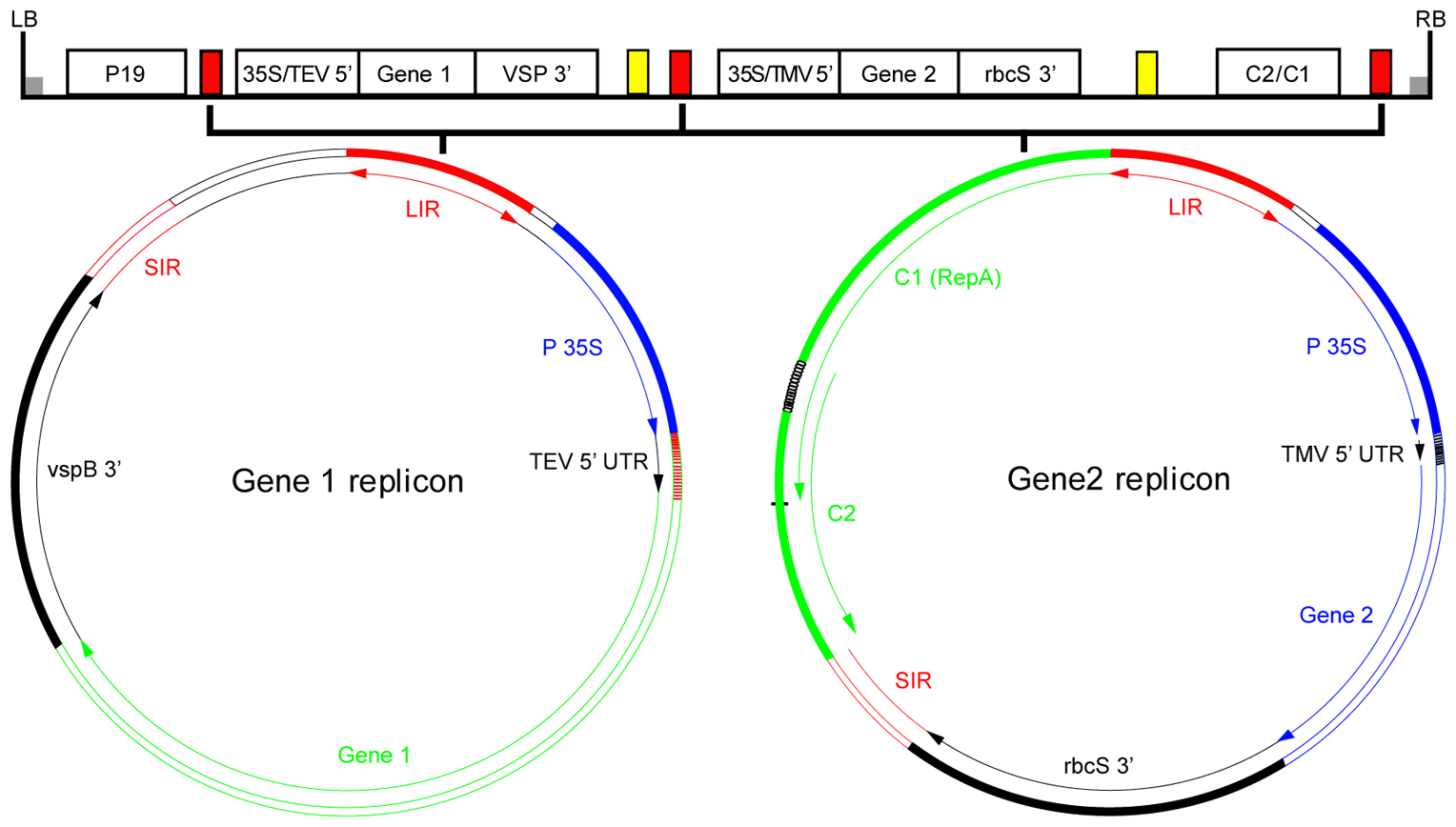
A single geminiviral BeYDV vector that can generate two non-competing replicons for co-expression of two proteins or one protein with two hetero-subunits. The left (LB) and right border (RB) delineate the T-DNA construct that will be transferred into plant cells by *Agrobacterium*. There are two gene constructs with each flanked by two LIR (red rectangles). Upon delivery into plant cells, expression of C1/C2 gene (next to the Gene 2 construct) produces the Rep protein that nicks the LIRs in the T-DNA to release two separate single-stranded DNA molecules. They are then copied to make double-stranded DNAs that can replicate by the rolling circle mechanism. The two replicons are amplified independently and non-competitively to produce high copy numbers of DNA templates and, in turn, abundant mRNAs for the translation of Protein1 and Protein 2. The expression of the gene silencing suppressor p19 further enhances the expression of both gene products. Yellow rectangles: SIR; LB: left border of the TDNA; RB: right border; p19: expression cassette for p19, a suppressor of gene silencing from TBSV; 35S/TEV5′: CaMV 35S promoter followed by tobacco etch virus 5′UTR; VSP3′: soybean vspB gene terminator; 35S/TMV5′: CaMV 35S promoter and TMV 5′UTR; rbsS3′: tobacco rbcS gene terminator; C2/C1 Rep/RepA gene [40].

Agroinfiltration can be performed with a variety of expression vectors, including non-viral or plant virus-based vectors [16]. While non-viral based vectors such as those driven by the 35S cauliflower mosaic virus (CaMV) promoter, can drive higher levels of protein accumulation in transient assays than in stable transgenic plants, the target protein yield is still relatively low [4]. On the other hand, vectors based on plant viruses can promote the accumulation of recombinant proteins to much higher levels due to their efficient replication and/or transcription in plant cells [3,22]. The earlier plant viral vectors were based on viruses with a double stranded DNA genome, such as CaMV. For example, the first successful expression of a transgene in plants was achieved by using a CaMV replacement vector, in which the insect transmission gene was replaced by a bacterial dihydrofolate reductase gene [23]. However, these viruses have very limited packaging capacity and easily lose their essential genome functions, even when only a small amount of their genomes are removed or substituted. As a result, these vectors have rigid constraints on the size of the transgene and have had limited impact on the advancement of the transient expression system. The next generation of viral vectors is based on single-stranded RNA viruses. The abundance and diversity of these viruses have allowed the identification of viral vectors that have a large packaging capacity and are more tolerable for gene substitution and insertion. For example, replacement vectors based on tomato bushy stunt virus (TBSV) and tobacco mosaic virus (TMV) have been used to express a variety of transgenes [3]. The viral coat protein (CP), which is essential for cell to cell movement of many viruses, is usually the target of gene replacement. The disadvantage of this strategy is the potential loss of systemic infectivity [3]. This is especially crucial for applications where infection of the entire plant is desired. This issue can be circumvented by the use of insertion vectors as they contain the complete functional viral genome with the addition of the target gene. These vectors, including those derived from TMV and potato virus X (PVX), allow the expression of the inserted transgene while retaining systemic movement and infection [3]. Traditionally, introduction of viral vectors to plant tissue is accomplished by mechanical inoculation of infectious viral particles or viral nucleic acids. This earlier method, however, faces several challenges in its application. For example, it cannot be used for viruses that are not susceptible to mechanical infection but require a specialized insect for transmission. For RNA virus-based vectors, it involves the laborious and hard-to-scale up *in vitro* process of generating RNA-based vectors. The limited host range of viruses also presents another obstacle for its broad application. The development and application of agroinfiltration have effectively resolved the problems associated with viral vector delivery. The cloned viral genomes in the DNA or cDNA form can be directly and efficiently delivered to the nucleus of plant cells by agroinfiltration without the need for the tedious *in vitro* transcription process. Accompanying the transcription and processing activities of the host cell, infectious and autonomously replicating nucleic acid constructs are produced from the delivered DNA [3]. The ability to deliver vectors to a majority of the cells on the entire plant by agroinfiltration also eliminates the need for the viral systemic spreading function. As a result, CP can be deleted to provide more options for the type and size of the transgene. In addition, the concern of transgene loss during systemic spreading is eliminated. Agroinfiltration also allows the application of viral vectors to a broad range of plant species beyond the natural virus hosts and to those that are not mechanically transmissible in nature. A recent fascinating development is in the area of “deconstructed” viral vectors. In this new generation of vectors, viral genome components which are not essential or beneficial for the function of an expression vector are eliminated. The deconstruction effectively reduces the size of the replicon to accommodate the insertion of larger transgenes, while maintaining the robustness of viral replication and transcription. The MagnICON system is one of the popular deconstructed viral vectors. This system is based on *in planta* assembly of replication-competent TMV and PVX genomes from separate pro-vector cDNA modules [24,25]. The 5′ module carries the viral RNA dependent RNA polymerase and the movement protein (MP), and the 3′ module contains the transgene and the 3′ untranslated region (UTR). *A. tumefaciens* strains harboring the two modules, along with a third construct that produces a recombination integrase, are mixed together and co-infiltrated into plant cells. Once expressed, the integrase assembles the 5′ and 3′ modules into a replication-competent TMV or PVX genome under the control of a plant promoter [25,26]. This assembled DNA construct is then transcribed and spliced to generate a functional infective replicon. In fact, the advantages of three biological systems are effectively integrated in the MagnICON system [24–27]. First, the use of *Agrobacterium* for vector delivery eliminates the complicated *in vitro* process of generating RNA vectors. Second, the risk of creating functional infectious particles is removed due to the deletion of CP genes, while the speed and high protein yield of the viral system is retained. Finally, this system integrates the posttranslational processing capacity of eukaryotic plant cells for producing complex proteins. Along with others, our laboratory has expressed numerous proteins from small subunit vaccines to large immune complexes with this system [5,6,28,29]. The collective results have demonstrated that this system is robust and drives high levels of target protein accumulation up to 1 mg per gram of leaf fresh weight (LFW) within 7 to 10 days after agroinfiltration [5,6,24,28,29]. This system retains the speed and expression amplification of viral vectors while gaining the flexibility of nuclear gene expression. Another prominent “deconstructed” viral vector is the geminiviral DNA replicon system derived from bean yellow dwarf virus (BeYDV) [12]. We developed this system primarily to resolve the difficulty in efficiently expressing multiple hetero-subunit proteins with other deconstructed viral vectors. This difficulty is caused by the phenomenon of “competing replicons”, as co-delivery of viral vectors built on the same virus backbone tends to result in early segregation and subsequent preferential amplification of only one of the vectors in a single cell [30–32]. The MagnICON system allows the production of monoclonal antibodies (mAbs) which contains two hetero-components: the light (LC) and heavy chain (HC). This is possible because TMV and PVX are non-competing viruses and allow the expression of both HC and LC and their assembly in a single plant cell [24]. However, it will be a difficult task to identify additional viruses compatible with both TMV and PVX for efficient expression and assembly of target proteins with three or more distinct subunits. Thus, there was a need to develop vector systems that allow efficient expression and assembly of multiple subunit proteins. BeYDV is a Mastrevirus of the *Geminiviridae* family and has a single-stranded circular DNA genome that can replicate to very high copy numbers by a rolling circle mechanism [33]. The genome of this virus consists of a long intergenic region (LIR), a short intergenic region (SIR), and four open reading frames that encode four proteins: the MP, the CP, and replication associated proteins, Rep and RepA [12]. Studies have demonstrated that only two *cis* acting elements (LIR and SIR) and one single viral protein (Rep) are required for viral replication [12]. Furthermore, the efficiency of viral replication is unaffected when the Rep is supplied in trans [12]. Correspondingly, we constructed the BeYDV geminiviral vectors only using the LIR and the SIR with the transgene cassette inserted between them, but without the MP and CP gene [12,20]. In the earlier version of our system, the gene for the Rep protein is provided in a second module [20]. Our results indicate that co-agroinfiltration of the transgene and Rep modules into plant cells results in robust replication of the transgene-carrying replicon and high-level accumulation of the recombinant protein [20]. We also demonstrated that the inclusion of another module harboring P19, a suppressor of gene silencing from TBSV, further enhances the target protein accumulation [20]. In the later version of this system we integrated the transgene, the Rep and P19 modules into a single vector system, which greatly simplifies the agroinfiltration process, especially for large-scale operations [21]. We further demonstrated that the BeYDV geminiviral system is non-competing and allows the efficient expression and assembly of mAbs [21]. Our research also indicates that multiple replicon cassettes encoding for different proteins/subunits can be incorporated into one single vector without compromising the expression levels of any transgene (Figure 2) [20,21]. While the yield of transgene products is comparable to that of the MagnICON system, the production time of the geminiviral system is slightly shorter as optimal accumulation of protein is usually observed within 4–8 days post agroinfiltration [20,21,34]. The geminiviral system may also have a broader plant host range than the MagnICON system as demonstrated by our studies with lettuce [34]. Overall, our single vector geminiviral replicon system not only provides quick and high-yield production capacity, but also allows for the possibility of producing proteins with up to 5 heterosubunits. Furthermore, it obviates the need for co-infection of multiple expression modules. Therefore, this system represents a significant advancement in transient expression technology for commercial production of pharmaceutical proteins.

## Growing Plant Material for Agroinfiltration

Unlike strategies using stable transgenic plants, transient expression systems do not generate transgenic lines and only require non-transgenic plant materials for agroinfiltration. Consequently, it does not bear the potential risk of food contamination or unwanted transgene outflow from genetically modified (GM) plants to non-GM crops or their wild relatives. This greatly reduces the regulatory and public acceptance issues for this technology. Our laboratory and others have extensively employed agroinfiltration for the transient expression of pharmaceutical proteins in several plant species (Table 1). While agroinfiltration can be performed on most plant materials, we found that the specific condition of plant material is the most likely factor in causing variability of recombinant protein yield between production batches and between laboratories [5]. In fact, the physiological state and the developmental stage of plants significantly influence their competency in producing the target protein through agroinfiltration. Our studies indicate that temperature, light intensity, supply of fertilizer, plant inoculation age, and incubation time after leaf infiltration are critical parameters for optimal plant growth and protein expression. The amounts of light, water and fertilizer available to plants must be consistent as minor changes in these conditions may drastically change the final size of plants and their ability to express recombinant proteins. For example, we found that growth under natural light produces much higher leaf biomass, but the yield of recombinant protein from this biomass is lower than that from leaves grown under artificial light [5]. Furthermore, our research demonstrates that the optimal condition to grow *Nicotiana benthamiana* plants is a 16 hr Light/8 hr dark cycle at 25 ± 0.5°C under such artificial lighting [5]. We also reported that 6-week old plants grown under these conditions yield the optimal biomass for the expression of the proteins we tested, including mAbs and virus-like particle (VLPs) vaccines. These plants have already accumulated sufficient biomass and consistently produce high-levels of target proteins. While plants older than 6-weeks produce more biomass, they have already initiated flower development which compromises the accumulation of recombinant proteins [5]. Furthermore, these plants are too tall to fit into the infiltration chamber or growth shelves, which are stacked to have the optimal space usage for maximal biomass production per cubic meter [5]. Therefore, 6-week old plants represent the optimal leaf material that balances the need of biomass yield, protein accumulation, space efficiency, and the ease of agroinfiltration. It is important to note that the conditions we reported above are specifically developed for *N. benthamiana* plants for infiltration with *Agrobacterium* harboring MagnICON and geminiviral vectors. If different host plant species or expression vectors are used, plant growth conditions will need to be re-optimized empirically. The most common host plants for transient expression of proteins are tobacco and the related *N. benthamiana,* because they are amenable to agroinfiltration, can produce high-yields of biomass rapidly, and are prolific seed producers for scale-up production [2,4]. In addition, a variety of expression vectors are already available for these species, providing another advantage. However, most tobacco and related *Nicotiana* plants contain high levels of phenolics and toxic alkaloids, which complicate the downstream purification of pharmaceutical protein production [35,36]. Our laboratory explored the feasibility of using lettuce (*Lactuca sativa*) as an alternative plant host to produce pharmaceutical proteins by agroinfiltration with deconstructed viral vectors. While lettuce can accumulate large amounts of biomass rapidly like tobacco, it produces negligible quantities of phenolics and alkaloids, and thus, would simplify the protein purification process. Our studies with two groups of protein candidates of pharmaceutical importance, namely VLPs and mAbs, indicate that lettuce is an excellent host for agroinfiltration with deconstructed viral vectors. The results showed that agroinfiltration with geminiviral replicon vectors permits high-level production of VLPs derived from the Norwalk virus capsid protein (NVCP) and therapeutic mAbs against Ebola (EBV) or West Nile (WNV) viruses in lettuce [34]. These vaccine and therapeutic mAb candidates can be readily purified in high-yield from lettuce leaves without the extra steps required for elimination of plant secondary metabolites. These lettuce-produced pharmaceutical agents not only are produced in large quantities, but also exhibit fully functional activity [34]. Significantly, our feasibility studies with grocery store-purchased lettuce also opened a new avenue for obtaining biomass for commercial scale agroinfiltration. Since lettuce is already grown in commercial greenhouses for mass production and biomass processing technologies are already in place, this allows us to have access to potentially unlimited quantities of inexpensive plant material for large-scale pharmaceutical protein production with agroinfiltration. The robustness and scalability of our geminiviral expression system, coupled with the virtually unlimited nature of commercial lettuce material generation, provide an agroinfiltration-based platform that is effective, safe, low-cost and scalable for commercial manufacturing.

## Preparation of *A. tumefaciens* Cultures

Preparation of the optimal culture of *A. tumefaciens* is another crucial step of agroinfiltration [37]. For transient expression of pharmaceutical proteins with either geminiviral or MagnICON vectors *via* agroinfiltration in *N. benthamiana* or lettuce, the following example process we developed has proven successful. *A. tumefaciens* strains containing the deconstructed vectors are first streaked from frozen stock on plates containing the appropriate antibiotics and grown over a period of 48 hours to obtain single colonies. An individual colony of each *Agrobacterium* strain is then selected and inoculated into a culture tube containing YenB media with the appropriate antibiotics [37]. The cultures are grown overnight at 30°C with a 300 RPM rotation rate. The OD_600_ values of each culture are recorded the next day and are used to calculate the necessary volume to start a larger subculture with a starting OD_600_ of 0.02. The new subcultures are allowed to grow at 30°C until the OD_600_ value is in the range of 1.7–2.0. Based on the particular OD_600_ value of each strain, an appropriate volume of *Agrobacterium* cells is then taken from the overnight culture to give a final OD_600_ of 0.12 for each strain in an infiltration mixture. *Agrobacterium* cells from each strain are first spun down at 12,000×g for 2 min and then resuspended in MES infiltration buffer [37]. The appropriate resuspended strains for target protein expression with either geminiviral or MagnICON vectors are then mixed, spun down again, and resuspended in MES infiltration buffer. In all above infiltration combinations, the final concentration of each strain is OD_600_=0.12 in MES buffer. The culture volume can be easily scaled up from a few milliliters for syringe infiltration, to several liters for pilot-scale vacuum infiltration, or even to thousands of liters for commercial scale vacuum infiltration. The most critical aspect of this process is the control of growth and infiltration concentration of *A. tumefaciens*, as measured by the OD_600_. Our research results indicate that it is important to grow each *A. tumefaciens* strain to, but not over the designated OD_600_ in each of the culture and subculture step. We have examined multiple concentrations of *A. tumefaciens* for the final agroinfiltration of *N. benthamiana* leaves [5,37]. Our results demonstrate that OD_600_=0.12 per *Agrobacterium* strain is the optimal concentration as it meets the needs for maximum transgene delivery without the risk of causing tissue necrosis and cell death [37,38]. For example, if a lower *Agrobacterium* concentration is used during agroinfiltration, it will result in low copy numbers of the target gene being delivered into cells, leading to low protein expression. If the concentration of *Agrobacterium* is too high, leaf necrosis will occur as a hypersensitive response in the infiltrated tissue is triggered [38]. Our results demonstrated that the desired OD_600_ density of *Agrobacterium* can be obtained by using consistent culture media, temperature and culture time.

## Agroinfiltration

**Figure 3:**
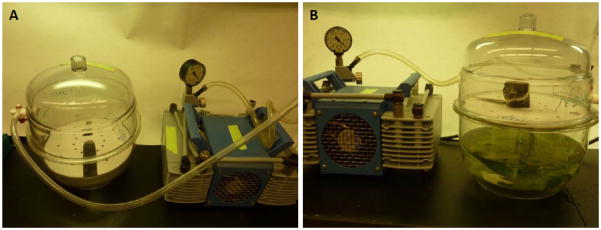
Vacuum agroinfiltration of *N. benthamiana* leaves with *Agrobacterium tumefaciens. A. tumefaciens* containing target gene construct was resuspended in infiltration buffer and loaded into a desiccator that was connected to a vacuum pump (A) The entire leaf system of a 6-week old plant was then submerged into the infiltration buffer (B). Agroinfiltration was achieved by applying and releasing a vacuum through the pump.

**Figure 4:**
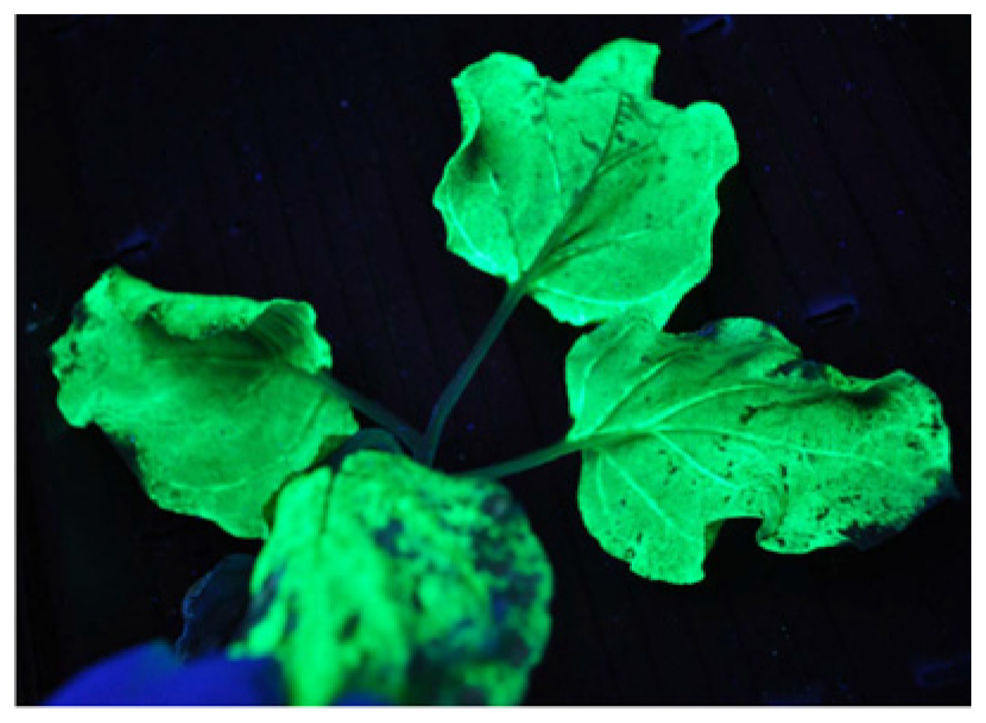
Expression of the green fluorescent protein in vacuum agroinfiltrated leaves. *N. benthamiana* leaves were infiltrated with *A. tumefaciens* harboring the GFP gene in MagnICON vectors. Leaves were photographed in a dark room under UV light 6 days post agroinfiltration [41,42].

**Figure 5:**
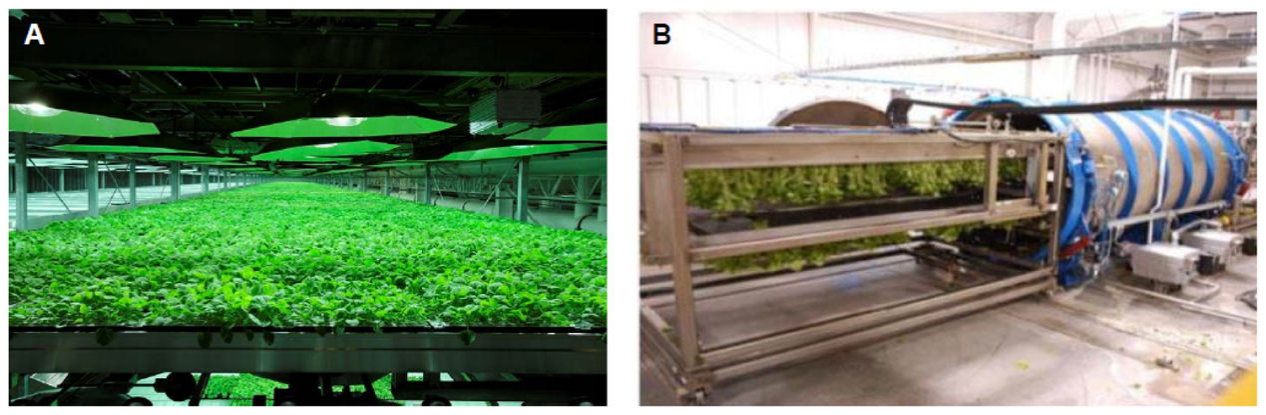
Commercial scale *N. benthamiana* plant growth (A) and agroinfiltration (B). (The photographs in this figure are kindly provided by Mr. Barry Bratcher of Kentucky Bioprocessing, LLC.).

Both syringe and vacuum infiltration have their applications for producing pharmaceutical proteins by transient expression. The simplicity of syringe infiltration allows a quick assessment on the expression level of the pharmaceutical candidate under established conditions. If the initial results are not ideal, the flexibility of conducting multiple assays on a single leaf will permit the further optimization of expression parameters. For example, different concentrations of *Agrobacterium* culture can be tested on a single leaf to compare their impact on the yield, expression kinetics and toxicity of the target protein. Similarly, the ability of different expression vectors in driving the target protein accumulation and their expression kinetics can be characterized. Other parameters, such as growth conditions, the organelle favorable for target protein accumulation, and the requirement for a silencing suppressor can be quickly established or identified by assays with syringe infiltration. Once these conditions are optimized for a new protein candidate, syringe infiltration can also be used to infiltrate the entire leaf area of several plants to rapidly obtain sufficient recombinant protein for its biochemical characterization, preclinical functional studies, as well as for developing its purification schemes [5,6]. The limitation of the syringe infiltration is its poor scalability. To develop an agroinfiltration-based transient expression platform applicable for commercial pharmaceutical protein production, a scalable agroinfiltration technology must be established. The potential scalability of vacuum infiltration makes it a prime candidate for this purpose. Our laboratory has established a vacuum infiltration procedure at the laboratory scale and examined its scalability in several studies with both VLP and mAb based pharmaceutical candidates [5,6,37]. We use a desiccator connected to a vacuum pump to provide the vacuum for infiltration (Figure 3A). Plants are placed upside down on a shelf and leaves are submerged into the infiltration media containing *Agrobacterium* (Figure 3B). A vacuum at 100 mbar is applied for 1 min, and the release valve is slowly opened to release the vacuum. Agroinfiltration is achieved by the exposure of the plants to the vacuum. The air in the interstitial space of the leaves is drawn out by the vacuum, and *Agrobacteria* in the infiltration medium enter and replace the air in this space when the vacuum is being released. Results from our research show that the accumulation level and the temporal expression pattern of target proteins are not altered by the switch from syringe to vacuum infiltration [5,6,20,21,28]. In fact, as the entire shoot of a plant is subjected to infiltration with the vacuum method, expression of the target protein can be detected over the entire plant, as illustrated by the GFP marker protein (Figure 4). Our research also indicates that vacuum infiltration is more robust and can significantly shorten the timeframe required for the infiltration of each plant. For example, syringe infiltration of a 6-week old *N. benthamiana* plant takes 15 min for a skilled worker to accomplish. In contrast, up to six plants can be vacuum infiltrated within 3 min from start to finish [37]. Our laboratory has carried out several scale-up studies using the vacuum infiltration for the production of pharmaceutical proteins (Table 2) [5,6]. To facilitate these studies, we designed a vacuum tunnel that is able to accommodate 16 trays of plants per infiltration cycle with automation [5]. These studies demonstrated that vacuum infiltration is not only highly scalable, but the temporal expression pattern and the levels of pharmaceutical proteins, such as NVCP VLPs and anti-WNV mAbs, produced under these scale-up conditions are similar to those obtained in smaller bench-scales studies [5,6,20,28]. Moreover, this technology is being explored by biotechnology companies to deliver transgenes on a manufacturing scale. For example, vacuum infiltration was attempted commercially to process several metric tons of *N. benthamiana* plants per hour by Kentucky Bioprocessing, LLC (Figure 5) [37]. To achieve consistent infiltration of plant material, it is important to not deviate from the vacuum pressure and vacuum duration optimized for each plant species at a specific infiltration scale. Indeed, the scale-up vacuum infiltration has allowed us to produce gram level of pharmaceutical grade of NVCP VLPs under current Good Manufacture Practice (cGMP) guidelines, sufficient both in quality and quantity for a phase I human clinical trial [4,5].

Thus, our studies also demonstrated the regulatory compliance of agroinfiltration for manufacturing human pharmaceuticals, as this technology can be operated under cGMP regulations of the US Food and Drug Administration (FDA). Collectively, these results indicate the strong feasibility of using agroinfiltration as part of the transient expression platform for producing human pharmaceuticals in plants.

## Conclusion

Plants have increasingly become an important host for manufacturing commercial pharmaceutical proteins with superior scalability, safety, speed, and cost-saving benefits. However, a persistent criticism of plants as a production platform of biologics has been the lack of plant-made human products approved by the FDA. This skepticism has been eliminated by the recent FDA approval of a plant cell-produced therapeutic enzyme for Gaucher’s disease [4]. This milestone heralds a new era for plant-made pharmaceuticals. The transient expression system has become the preferred plant-based platform due to its advantage in protein production yield and speed, as well as the lack of concern for transgene escape and contamination of food crops. The development of novel expression vectors, especially those based on deconstructed plant viruses by our laboratory and others, has further solidified the feasibility of the transient expression system as a production platform. These vectors have significantly enhanced accumulation levels of pharmaceutical proteins and allowed plants to compete with mammalian cell-based platforms. One of the last remaining hurdles for the commercial application of plant transient systems was the lack of a scalable technology to deliver transgene into plant cells. As discussed in this review, agroinfiltration based on syringe and vacuum infiltration has overcome this challenge and provides an efficient, robust, and scalable gene-delivery technology for the transient expression of recombinant proteins in plants. Along with deconstructed viral vectors, it will greatly facilitate the implementation of plant transient expression systems as a premier commercial platform for the rapid, economical and safe production of pharmaceutical proteins.

## Figures and Tables

**Table 1:** Examples of plant-derived pharmaceutical proteins produced via agroinfiltration.

Plant Host	Pharmaceutical Target	Development Stage	References
*N. benthamiana*	Influenza A H5N1 HA VLP vaccine	Phase I/II clinical trial	[39,40]
*N. benthamiana*	Influenza A H1N1 HA VLP vaccine	Phase I	[41]
*N. benthamiana*	Ebola immune-complex vaccine	Preclinical	[29]
*N. benthamiana*	HBcAg non-enveloped VLP vaccine	Preclinical	[20]
*N. benthamiana*	HBsAg enveloped VLP vaccine	Preclinical	[4]
*N. benthamiana*	HIV-1 Pr55gag vaccine	Preclinical	[4]
*N. benthamiana*	WNV prM/M and E vaccine	Preclinical	[4,12]
*N. benthamiana, Lettuce*	Ebola therapeutics based on mAb	Preclinical	[21,28,34,42]
*N. benthamiana, Lettuce*	Norovirus NVCP VLP vaccine	Preclinical	[18,20,34]
*N. benthamiana, Lettuce*	WNV therapeutics based on mAb	Preclinical	[6,34]
*N. benthamiana, Lettuce*	WNV DIII vaccine	Preclinical	[12,34]

HA: hemagglutinin; VLP: virus-like particle; HBcAg: hepatitis B core antigen; HBsAg: hepatitis B surface antigen; HIV: human immunodeficiency retrovirus; Pr55gag: major core protein Gag precursor; WNV: West Nile virus; PreM/M: premembrane and membrane protein; E: envelope protein; mAb: monoclonal antibody; NVCP: Norwalk virus capsid protein; DIII: domain III of envelope protein.

**Table 2:** Examples of agroinfiltration scalability studies by our laboratory.

Target protein	Leaf Biomass (g)	Protein Yield (μg/g LFW ± SD)	Reference
NVCP VLP vaccine	100	420 ± 34.24	[5]
NVCP VLP vaccine	500	402 ± 52.03	[5]
NVCP VLP vaccine	15,000	418 ± 46.42	[5]
WNV mAb therapeutic	10	727 ± 87.36	[6]
WNV mAb therapeutic	100	740 ± 101.49	[6]
WNV mAb therapeutic	500	697 ± 90.74	[6]
WNV mAb therapeutic	5,000	598 ± 68.06	[6]

LFW: leaf fresh weight; SD: standard deviation; NVCP: Norwalk virus capsid protein; VLP: virus-like particle; WNV: West Nile virus; mAb: monoclonal antibody.
